# Pancytopenia Misattributed to Parvovirus B19 Infection Revealing Acute Promyelocytic Leukemia

**DOI:** 10.7759/cureus.115747

**Published:** 2026-09-03

**Authors:** Florine Bachmann, Fiona El Khazen, Nicolas Garin, Dina Milowich

**Affiliations:** 1 Internal Medicine, Hôpital Riviera-Chablais, Rennaz, CHE; 2 Histopathology, Hôpital Riviera-Chablais, Rennaz, CHE

**Keywords:** acute myeloid leukemia (aml), acute promyelocytic leukemia (apl), bone marrow biopsy, pancytopenia, parvovirus b19

## Abstract

Pancytopenia is a diagnostic challenge due to its broad differential diagnosis, including infections and hematological malignancies. Parvovirus B19 infection is a recognized but uncommon cause of transient pancytopenia. However, positive viral serology or viremia may lead to diagnostic anchoring and delay the identification of underlying malignant disorders. We report the case of a 17-year-old previously healthy female presenting with persistent fever and tonsillitis. Laboratory investigations revealed pancytopenia with agranulocytosis. Blood serology and polymerase chain reaction (PCR) were positive for Parvovirus B19. The peripheral blood smear was normal. An initial diagnosis of virus-related pancytopenia was established. Despite supportive treatment, including granulocyte colony-stimulating factor, transfusions, and intravenous immunoglobulins, pancytopenia persisted. Bone marrow aspiration and biopsy were performed. The bone marrow aspiration showed a hypercellular marrow without abnormal cells by flow cytometry within the conventional blast gate. Subsequent pathological examination of the biopsy revealed massive blast infiltration consistent with acute promyelocytic leukemia. The patient rapidly developed disseminated intravascular coagulation, and atypical promyelocytes later appeared in the peripheral blood smear. Targeted treatment with all-trans retinoic acid and arsenic trioxide was initiated, leading to remission. This case highlights the risk of delayed diagnosis when pancytopenia is attributed to viral infection. Persistent cytopenia, even in the presence of positive Parvovirus B19 testing and a normal blood smear, should prompt early bone marrow examination to exclude underlying hematological malignancy.

## Introduction

Pancytopenia is a challenging hematological condition due to its broad differential diagnosis ranging from benign to potentially fatal diseases. Initial evaluation includes a complete blood count with reticulocytes, coagulation tests, liver and kidney function tests, and a peripheral blood smear to assess for possible hematologic malignancy. Further investigations should be guided by the patient's history and a physical examination [1].

Acute myeloid leukemia (AML) and myelodysplastic syndromes (MDS) are examples of malignant causes of pancytopenia. Acute promyelocytic leukemia (APL) is a distinct subtype of AML characterized by the t(15;17)(q24;q21)/PML::RARA translocation [2]. Although it accounts for only approximately 10% of AML cases, it constitutes a hematological emergency because untreated disease is rapidly fatal, mainly due to disseminated intravascular coagulation (DIC) and severe hemorrhage [3]. Prompt recognition is therefore essential, as early initiation of differentiation therapy markedly improves survival. Peripheral blood smear abnormalities, such as macrocytosis, circulating blasts, or leukoerythroblastosis, may suggest a hematological malignancy, but a normal peripheral blood smear does not exclude it [4]. Moreover, in APL, particularly the hypergranular subtype, circulating leukemic cells may be absent or very scarce despite extensive bone marrow infiltration, making diagnosis on peripheral blood examination particularly challenging. Therefore, bone marrow examination remains the gold standard for diagnosis. Complications of bone marrow aspiration and biopsy are rare, even in the setting of pancytopenia [2].

Primary Parvovirus B19 (PVB 19) infection is common and is usually asymptomatic or presents with a self-limited influenza-like illness followed by rash in children or arthralgia in adults. The seroprevalence increases with age, 20-40% between five and 18 years old, 40 to 60% in adults [5]. Although PVB 19 primarily affects the erythroid lineage, particularly in patients with underlying hematological disorders, it may exceptionally cause transient pancytopenia in patients without underlying hematological disorders. Therefore, persistent or worsening pancytopenia despite a diagnosis of acute PVB 19 infection should prompt investigation for an underlying bone marrow disorder. Because acute PVB 19 infection is common whereas APL is rare but immediately life-threatening, attributing pancytopenia solely to documented PVB 19 infection may delay the diagnosis of an underlying hematologic malignancy. Reports of this diagnostic overlap remain uncommon, making awareness of this potential pitfall clinically important.

We report a case of pancytopenia initially attributed to acute PVB 19 infection based on clinical findings and positive serology and viremia, with a normal peripheral blood smear. However, bone marrow biopsy ultimately revealed APL, demonstrating the complexity of the differential diagnosis of pancytopenia and the importance of bone marrow examination when the clinical course is not rapidly favorable despite an apparently plausible infectious diagnosis.

## Case presentation

A 17-year-old previously healthy female presented with odynodysphagia, dysphonia, and fever that had started seven days prior. Physical examination revealed white exudates on the right tonsillar pillar. Laboratory tests showed pancytopenia with aregenerative anemia, leukopenia with agranulocytosis, and thrombocytopenia (Table 1). A computed tomography (CT) scan of the neck showed phlegmonous infiltration of the right tonsil with an incipient submucosal abscess (Figure 1). The patient was admitted to the internal medicine department, and treatment was initiated with piperacillin-tazobactam and corticosteroids. The pancytopenia was initially attributed to consumption in the setting of acute bacterial infection.

**Table 1:** Laboratory results of the patient during hospitalization.

Test	Day 0	Day 2	Day 5	Day 12	Day 14	Reference range
Hemoglobin (g/L)	98	104	93	79	70	117-157
Reticulocytes (G/L)	24	-	-	22	-	50-120
Leukocytes (G/L)	0.8	3.1	5.2	2.6	3.9	4-10
Neutrophils (G/L)	0.2	1	0.1	0.2	0.4	1.6-7.5
Thrombocytes (G/L)	91	18	13	21	10	150-350
Prothrombin time (%)	92	83	-	88	57	70-140
Partial thromboplastin time (seconds)	30	18	-	28	27	25-36
Fibrinogen (g/L)	-	2.7	-	-	1.9	1.8-4
D-dimers (ug/L)	-	-	-	-	>35000	<500
Folate (nmol/L)	-	1.6	-	-	-	8.8-60.73

**Figure 1:**
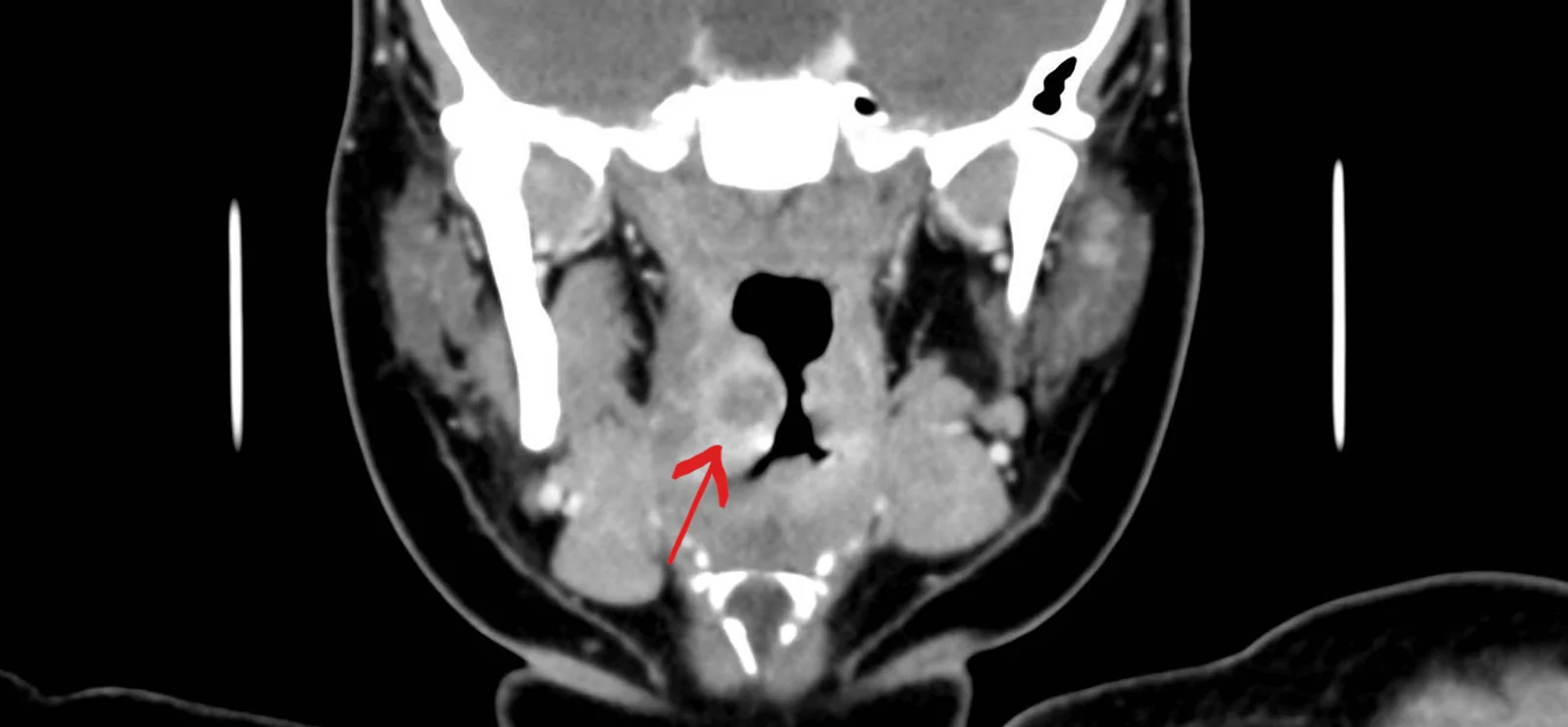
Coronal section of a CT scan showing a phlegmonous infiltration of the right tonsil with a submucosal abscess (red arrow).

The clinical course was rapidly favorable, with resolution of fever and tonsillitis, as well as improvement in leukopenia but worsening of thrombocytopenia. Ancillary analyses revealed low folate level (Table 1), which was supplemented. Peripheral blood testing done on the first day of admission was positive for PVB 19 by polymerase chain reaction (PCR) (571 copies/mL). Pancytopenia secondary to acute PVB 19 infection was suspected. The patient received granulocyte colony-stimulating factor (G-CSF), packed red blood cells, platelet concentrates, and intravenous immunoglobulins (400 mg/kg). 

On day 6, the pancytopenia persisted. As PVB 19 serology was suggestive of previous infection (IgG > 150 kUl/L and negative IgM), the diagnosis of bone marrow suppression due to acute PVB 19 infection was questioned, and a bone marrow aspiration and biopsy were finally performed on day 12. The first examination of the bone marrow aspirate revealed abundant hematopoietic precursors in all three lineages, with a predominantly myeloid lineage. Flow cytometry on the bone marrow aspirate found no evidence of myeloid or lymphoid blasts. In view of these results, the diagnosis of pancytopenia due to PVB-19 infection was maintained. 

On day 14, the patient developed DIC characterized by elevated D-dimers, a reduced prothrombin time, a normal activated partial thromboplastin time, and decreasing fibrinogen level (Table 1). Physical examination revealed no evidence of bleeding. Atypical promyelocytic cells were visible for the first time on peripheral blood smear examination.

The laboratory was asked to perform further flow cytometry of the bone marrow as the granulocyte gate had not been analyzed. It revealed 48% medullary myeloid blasts with a CD13+, CD33+, CD38+, CD4+, CD34-, HLA-DR-, CD117+ phenotype.

Consequently, a diagnosis of APL was retained, and targeted treatment with all-trans retinoic acid (ATRA) and arsenic trioxide was administered. A few days later, the histopathological examination of the biopsy revealed a hypercellular hematopoietic marrow with 70% infiltration by immature blasts, consisting mostly of promyelocytes. They exhibited irregular, often indented nuclei along with eosinophilic granulations in the cytoplasm, as well as some Auer rods, and were diffusely positive for CD117 and myeloperoxidase, and negative for CD34 (Figures 2-3). Cytogenetic analysis showed the presence of a t(15;17)(q24;q21) translocation characteristic of APL using the optical genome mapping (OGM) technique.

**Figure 2:**
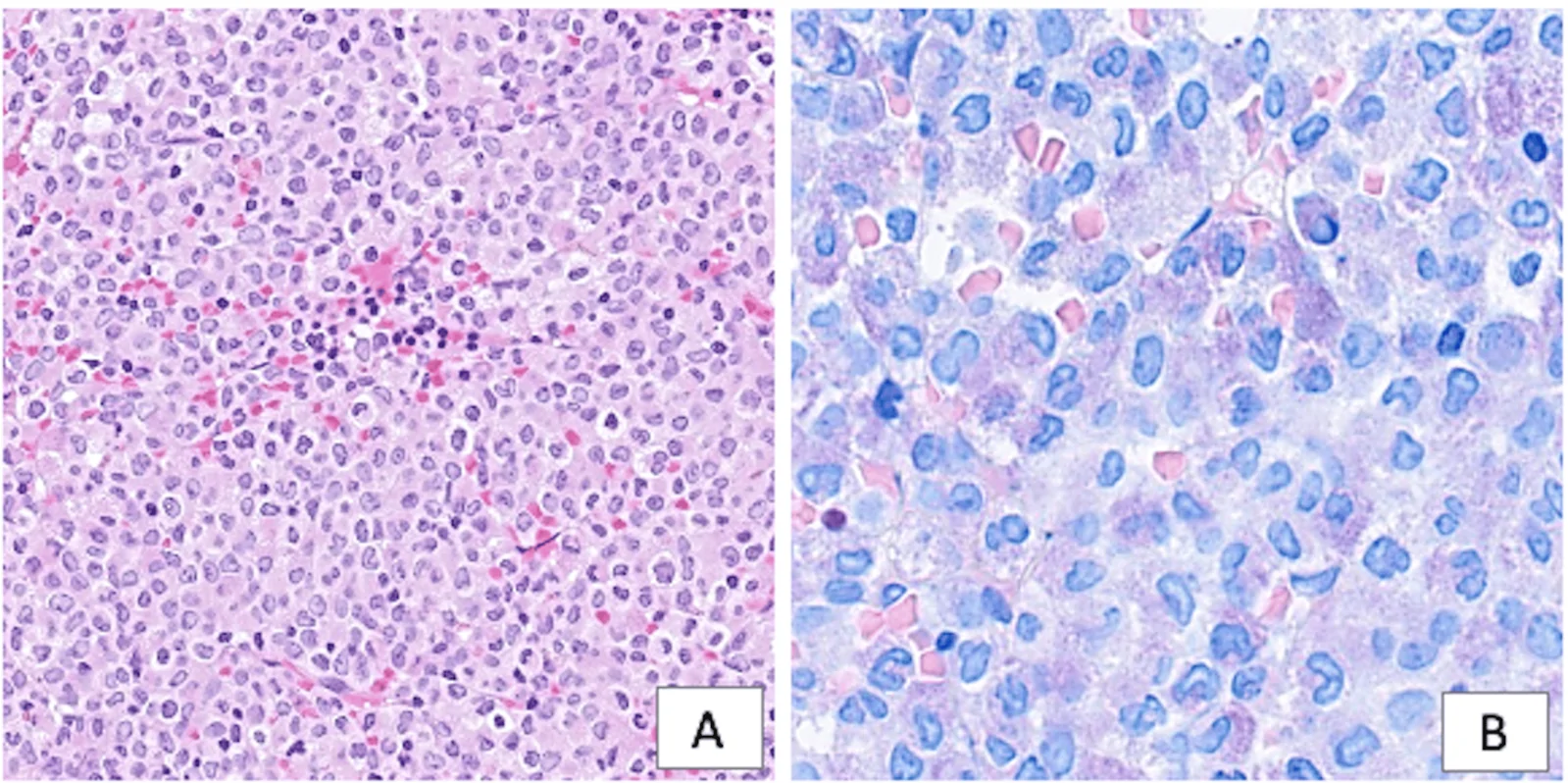
Bone marrow histology showing a diffuse infiltrate of atypical promyelocytes (A) with ovoid, bilobated, or reniform nuclei and prominent cytoplasmic granulations (B).

**Figure 3:**
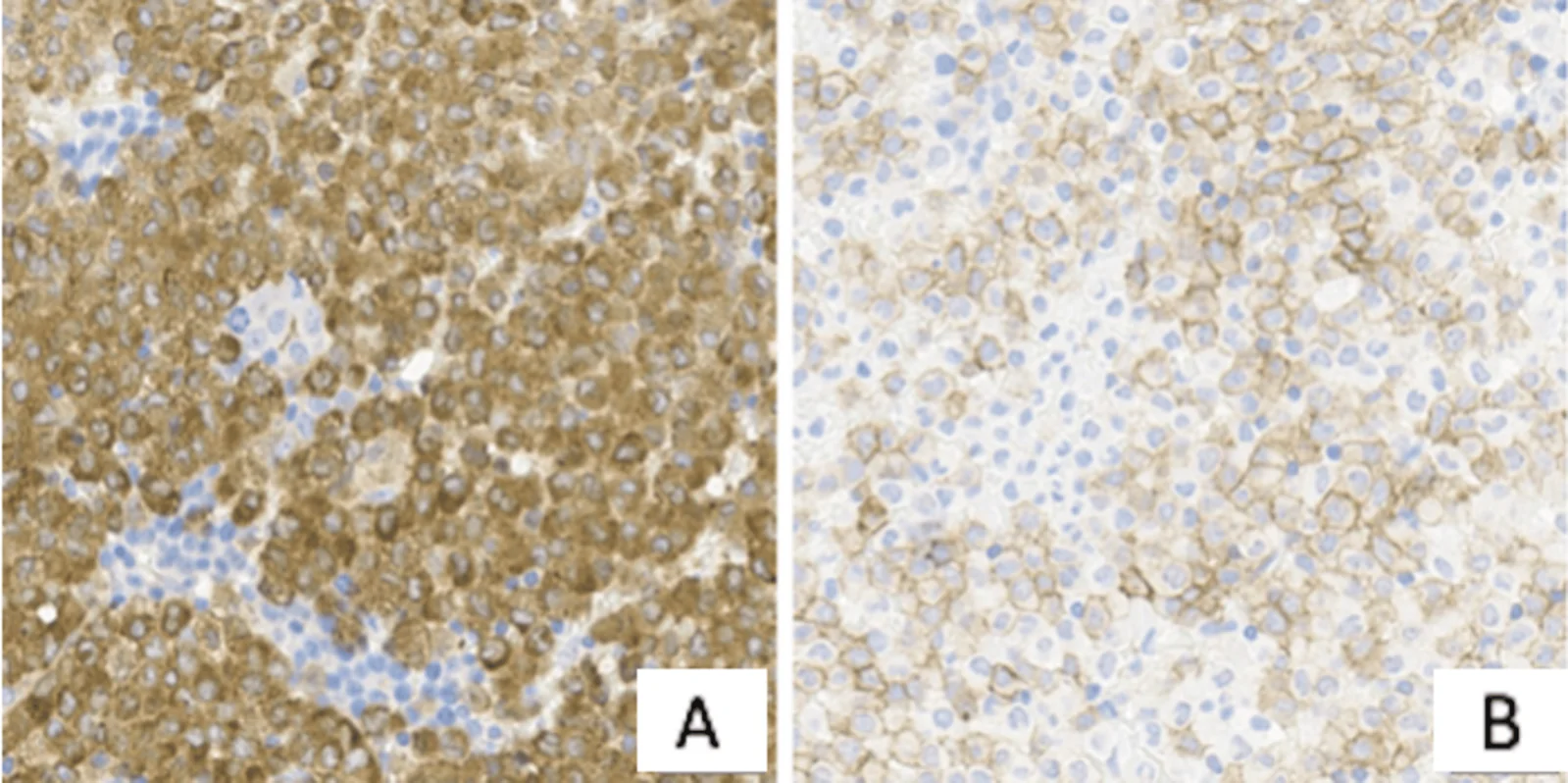
On bone marrow histology, the blasts were diffusely positive for myeloperoxidase (A) and CD117 (B) by immunohistochemistry.

The treatment was well tolerated and led to complete remission.

## Discussion

This case highlights the diagnostic challenge of pancytopenia due to the overlap between infectious and malignant hematological conditions. AML may present with non-specific symptoms related to pancytopenia, such as fatigue, fever, hemorrhagic diathesis, or a combination of symptoms that can mimic infection and delay the diagnosis when the patient tests positive for viral infection [5].

Rarely, PVB-19 infection can cause pancytopenia, which is most often transient and without clinical consequences, typically presenting at the onset of infection during the virological peak [5,6]. One hypothesis suggests that PVB-19-specific cytotoxic T lymphocytes, along with the viral NS1 protein, participate in the destruction of bone marrow precursors via the activation of cytokines such as tumor necrosis factor-alpha and interleukin-6, which are responsible for cytopenia. Furthermore, PVB-19 has a marked tropism for CD36+ human erythroid progenitors expressing the P antigen (globoside), leading to cell cycle arrest and cell death by NS1-mediated lysis or apoptosis [5,6].

The diagnosis of acute PVB-19 infection is based on serology with the detection of anti-VP2 IgM, which has high sensitivity but may remain positive for several months, and exceptionally beyond six months, after the acute infection [6-8]. Although high viral loads of PVB-19 correlate with clinical severity, particularly in acute erythroid aplasia in immunocompromised patients or those with blood disorders, viremia is not a reliable marker of the symptomatic phase because it may persist at low levels for months and even years after the infection without clinical significance [5-7]. A threshold of approximately 10⁴ copies/mL may help distinguish potentially symptomatic viremia from asymptomatic persistence [5]. Viremia remains useful in immunocompromised patients in whom serology may be altered or in the early phase (days 6 to 15) when seroconversion has not yet occurred [5-7]. In the event of serological discordance (isolated IgM with negative PCR), a follow-up serology test at two to six weeks demonstrating a significant increase in IgG can confirm recent infection [5]. In our case, the laboratory tests revealed a positive viremia, misleading for an acute infection; then the serology revealed the presence of IgG without IgM, which is consistent with an old infection. 

Some studies report a possible association between PVB-19 infection and hematologic malignancies, particularly AML, suggesting that the diagnosis of PVB-19 can precede the development of AML by 180 days or complicate the clinical presentation of leukemia [6].

Unlike other subtypes of AML, APL most commonly presents with leukopenia, and abnormal cells may be absent in the peripheral blood smear, possibly delaying diagnosis. In our case, the initial bone marrow flow cytometry was interpreted as normal, which likely also contributed to this delay. This can be explained by the fact that leukemic promyelocytes in classical hypergranular APL exhibit high side scatter (SSC), which indicates a highly granular or structurally complex cell, causing them to fall within the granulocyte region during flow cytometry, rather than in the conventional blast gate. A blast percentage reported as normal within the conventional blast gate does not exclude hypergranular APL if the granulocyte gate is not specifically interrogated [9]. Furthermore, APL is often accompanied by coagulopathy and may be complicated by DIC, which is associated with high mortality [10], particularly before or in the early phase of treatment. Prompt initiation of specific treatment (ATRA and arsenic trioxide) is critical and leads to complete remission in >80% of cases [11].

## Conclusions

This patient’s history illustrates the risk of delayed diagnosis when pancytopenia is attributed to viral infection. The presence of PVB-19 viremia associated with a normal blood smear should be interpreted with caution in the setting of persistent cytopenia, as it is an uncommon complication of acute PVB-19 infection in a patient without known hematological disorder. Although APL is rarer than PVB-19 infection, it is important not to miss the diagnosis because of its potentially fatal outcome. Therefore, in cases of unexplained pancytopenia persisting for more than a few days, bone marrow aspiration and biopsy should be considered to exclude underlying hematological malignancy. Bone marrow biopsy remains the gold standard for diagnosis, with complications being rare even in the setting of pancytopenia. Coagulation tests should also be monitored, as the emergence of signs of DIC should strongly raise the suspicion of APL.
